# Investigating the effects of COVID-19 lockdown on Italian children and adolescents with and without neurodevelopmental disorders: a cross-sectional study

**DOI:** 10.1007/s12144-021-02321-2

**Published:** 2021-10-25

**Authors:** Cristiano Termine, Linda Greta Dui, Laura Borzaga, Vera Galli, Rossella Lipari, Marta Vergani, Valentina Berlusconi, Massimo Agosti, Francesca Lunardini, Simona Ferrante

**Affiliations:** 1 Child Neuropsychiatry Unit, Department of Medicine and Surgery, School of Medicine, University of Insubria, Piazza Biroldi 19, 21100 Varese, Italy; 2 Department of Maternal and Child Health, Del Ponte Hospital, Varese, Italy; 3 Department of Electronics, Information and Bioengineering, NearLab, Politecnico Di Milano, Milan, Italy; 4 Child Neuropsychiatry Unit, Department of Clinical and Experimental Sciences, University of Brescia, Brescia, Italy; 5 Paediatric Unit, Department of Medicine and Surgery, University of Pavia, Pavia, Italy; 6 Paediatric Unit, Department of Medicine and Surgery, University of Insubria, Varese, Italy

**Keywords:** COVID-19, Neurodevelopmental Disorders, Tic, Autism Spectrum Disorder, ADHD, Specific Learning Disorders

## Abstract

We conducted a cross-sectional study to compare the impact of social distancing and lifestyle changes that occurred during Corona Virus Disease 2019 (COVID-19) lockdown on children and adolescents with and without Neurodevelopmental Disorders (NDDs). An online questionnaire was administered in order to investigate the effects of NDD condition, socio-demographic status, familiar/home environment and COVID-19 exposure on their lives during a two months period of social isolation. We used logistic regression, focusing on five endpoints (remote learning, lifestyle, stress/anxiety, sociality, scolding) to define the extent of these effects. Most questions were paired up to parents and children, to verify the occurrence of agreement. 8305 questionnaires were analyzed, 1362 of which completed by NDDs and 6943 by controls. Results showed that the presence of a NDD, compared to controls, had a significant impact on: *Remote Learning* (i.e. subjects with NDDs experienced more difficulties in attending online classes and studying), *Sociality* (i.e. subjects with NDDs missed their schoolmates less), S*colding* (i.e. subjects with NDDs were scolded more often) and *Anxiety* (i.e. subjects with NDDs were perceived by their parents as more anxious). Substantial agreement between parents and children arose from questions concerning *Remote learning*, *Lifestyle* and *Scolding*. The current study actually points out that having a NDD gives account for a stronger influence on school performance and on behavioral and psychological aspects, during a two months lockdown. Such results may provide useful information to governments and school authorities on how carrying through supportive strategies for youth affected by NDDs.

**Supplementary Information:**

The online version contains supplementary material available at 10.1007/s12144-021-02321-2.

## Introduction

The World Health Organization declared a public health emergency of international concern for the COVID-19 on 30^th^ January 2020. Italy was one of the most affected countries at the beginning of 2020 and COVID-19 outbreak was particularly dramatic in Lombardy (a northern region of Italy). In February 2020, Italian government applied mandatory rules for its citizens to abide by and social isolation was adopted as the main measure to prevent the spread of the viral infection (WHO, 2020). The lockdown thus included home confinement, interdiction of outdoor activities and indefinite closure of schools, rehabilitation and educational centers and sport associations. Such measures resulted in upheaval for citizens’ everyday life, and this was notable amongst families with school-aged children. Many parents had to shift to home-working, while they had to take care of their children because schools were closed. Childcare and homeschooling had to be managed without the usual supportive systems, such as school extra-services and therapists. Hence, parents often felt unprepared to carry out both home-working and child-caring successfully (Cluver et al., 2020). As for children, school closure was the most disruptive change in their routine. The school system quickly attempted to restore normality: alternative educational tools were introduced by developing distance learning solutions (United Nations, 2020). Both children and teachers tried to use the new educational models, but virtual communication was found to negatively affect the relationship between teachers and students previously created in the classroom, that is key to influencing student’s motivation (Khalilzadeh & Khodi, 2018). During lockdown, many life changes impacted children’s lives: school closure, lack of outdoor activities, aberrant dietary and sleeping habits. All these changes could promote distress and exacerbate any latent neuropsychiatric or psychological symptoms (Ghosh et al., 2020). Therefore, although children seemed to be the less affected by the virus, they were hit the hardest by the psychosocial consequences of social distancing (Golberstein et al., 2020). The negative consequences of the pandemic were certainly exacerbated when a pre-existent neuropsychiatric or psychological condition was present, such as in the case of Neurodevelopmental Disorders (NDDs). NDDs are a group of medical conditions that have their onset during childhood and are life-lasting; they result in functional limitations concerning neuropsychological, cognitive, and adaptive development. DSM-5 (American Psychiatric Association, 2013) considers neurodevelopmental disorders as a single cluster, which includes subjects affected by Autism Spectrum Disorder (ASD), Attention Deficit/Hyperactivity Disorder (ADHD), Specific Learning Disabilities (SLD), Tourette Syndrome (TS/Tics), intellectual disability and language (communication) disorders. Individuals with ASD typically present abnormalities in understanding the intent of others, displaying diminished interactive eye contact and atypical use and understanding of gesture (Hyman et al., 2020). ADHD is associated with deficits across a range of cognitive domains (e.g., executive functions, working memory) (Posner et al., 2020) so that such patients have great difficulties in following instructions and understanding complex situations, such as the pandemic. SLD subjects are identified by impairments in automatizing learning processes, such as reading, writing and/or math (American Psychiatric Association, 2013). TS/Tics subjects are characterized by sudden, involuntary, purposeless, and partially controllable movements and vocalizations/noises (Arzimanoglou et al., 2018). Intellectual disability includes, instead, deficits in cognitive and adaptive functions, such as language, memory, logical-mathematical and visuo-cognitive skills and organization of activities of daily living (American Psychiatric Association, 2013). All these conditions have "domains of difficulty" in common, although they can be compromised within various ranges of severity: emotional and behavioral issues, family stress, impaired learning, communication, and social skills (Thapar et al., 2017). Frequently, a child may have co-occurring diagnoses because the neural correlates of NDDs often encompass diffuse brain injury (Van den Berg et al., 2018) with consequent functional impairment in multiple domains. Thus, a multidisciplinary therapeutic approach is often adopted to treat neurodevelopmental disorders (Arzimanoglou et al., 2018).

Our hospital care experience during lockdown confirmed the great disadvantage of children affected by NDD: very often, their parents asked for specialistic support, after having noticed a worsening in behavioral symptoms, a reduced compliance with online school and a progressive change in their everyday lives. To reduce this negative impact, some neuropsychiatric units in Italy have promptly developed useful strategies (telerehabilitation, tele-consultation, telemedicine) (Fazzi & Galli, 2020). However, a comprehensive response to these needs could not be completely guaranteed during periods of social isolation; assessment and treatment for children with these disorders require the cooperation of specialists, many of whom are based in different services, which may be impossible to reach. To envisage a targeted support for these children, it is important to define the specific areas of difficulty to deal with. First, the proposed remote learning solutions proved harder to accomplish for students affected by NDDs. In fact, shifting classes online led to a dearth of special educational assistance to children, for at least two reasons: (i) parents could not completely manage special educational needs, because they have no specific professional training; (ii) a lack of assistive technologies was sometimes present. The most obviously disadvantaged groups are those with intellectual disabilities and SLDs, which may be particularly affected in their school outcomes during distance learning. In addition, the difficulty in coping with complex situations in children with ADHD led them into more troubles with respect to their peers in complying with remote learning, even in adolescence. This was confirmed by their parents, who realized—by spending more time together—the hard difficulties in both managing and supporting them (Becker et al., 2020). A second area to investigate is the degree of change in lifestyle habits caused by forced home confinement, that could have negatively impacted psychological stability. As an example, in ADHD patients, a recent study reported a disruption in everyday life, with a greater use of digital devices and changes in sleeping habits (Sciberras et al., 2020). Third, it is likely that COVID-19 pandemic resulted in increased stress and anxiety, given the fact that individuals with NDDs are particularly susceptible to distress caused by physical distancing measures. In children with intellectual disability, given their reduced adaptive functions, the COVID-19 pandemic exerted a psychological impact, including high levels of stress, anxiety, and depression, in both disabled children and their families. Patients with TS/Tics can be disadvantaged as well during a pandemic lockdown, a source of distress; in fact, the severity of tics is magnified under environmental and psychological pressures. A fourth critical consequence of lockdown was the decrease in social relationships, as social distancing was the first measure adopted to contain the spread of the virus. In children with intellectual disabilities, traumatic life events, like a pandemic, can intensify the feelings of social stigma and discrimination, already existing in these subjects. Sociality was disrupted in ADHD as well (Sciberras et al., 2020). In ASD subjects, the already occurring social difficulties are likely to be negatively impacted by the forced isolation due to the pandemic status. Finally, emotional and cognitive profiles of SLD subjects normally benefit from the cooperative relationships with classmates and teachers (Kiuru et al., 2020), that was greatly diminished by social distancing. In the end, a fifth area of difficulty faced during the COVID-19 lockdown was the potential deterioration of relationships also amongst family members, that were forced to spend all day together and to share resources. In this context, ADHD children might display increased behavioral problems (Cortese et al., 2020). Moreover, a recent study (Alhuzimi, 2021) reports how stress and lack of support in families with children affected by ASD can bring a deterioration in parental emotional wellbeing. On the other hand, familiar patterns of SLD children, especially those with dyslexia, are often anxiety-oriented and prone to conflict avoidance models (Bonifacci et al., 2014). Therefore, in this study we hypothesized that COVID-19 restrictions notably hurt NDDs subjects in different ways, since they usually depend on the support of caregivers and networks of services to maintain their emotional balance and mental health (Tajè et al., 2020). The aim of our study is to evaluate whether the COVID-19 restrictions had a greater impact in subjects with NDDs than in controls.

## Objective and Significance of the Study

This study has the ambition to guide decision makers towards a more targeted treatment for children and adolescents with NDDs in crisis situations, such as the COVID-19 lockdown. The assessment of pedagogical or psychological needs would produce a refinement in the intervention efficacy. Indeed, we defined the different areas in which children and adolescents experienced difficulties during lockdown (i.e. *Remote Learning, Lifestyle, Anxiety, Social* and *Scolding*) as potential areas of intervention. Therefore, the primary aim of this study was to identify which factors (demographical, environmental or pathological) impacted the most on each area of difficulty, with a special focus on the role of the presence of a NDD condition. The secondary aim was to investigate if parents and children/adolescents had the same perception of the effect of the lockdown on children/adolescents’ lives and whether this agreement changes for families having children with NDD. To reach these aims, we designed a questionnaire that was proposed at scale to students attending all grades of the school system, exploiting different data sources, such as hospitals and associations, but mainly the schools’ network. The latter was available thanks to the cooperation with the local school office, which allowed to reach as many students as possible, being potentially representative of most of the population. Our results could be helpful to implement information about NDD vulnerability in lockdown and could ideally be used to improve virtual help tools, not only in a future pandemic scenario, but also in addition to the traditional health and didactic resources.

## Methods

### Participants

As we aimed at achieving a comprehensive view of the lockdown effect on the school-aged population, we included in the study children and adolescents living in the province of Varese, attending a grade comprised between the first year of primary school and the fifth year of high school, together with their parents. It must be considered that the Italian school system is divided into primary school (PS, five grades—from 6 to 10 years), middle school (MS, three grades—from 11 to 13 years) and high school (HS, five grades—from 14 to 19 years). To recruit a large and representative sample of parents and children, different sources were leveraged: 1) Child Neuropsychiatry Unit of Filippo Del Ponte Hospital in Varese; 2) advocacy associations for people with different NDDs: AIFA (Associazione Italiana Famiglie ADHD – Italian association of ADHD families), ANGSA (Associazione Nazionale Genitori Soggetti Autistici – National association of ASD parents) and AIST (Associazione Italiana Sindrome di Tourette – Italian association of Tourette syndrome); 3) websites of schools in the province of Varese. An online questionnaire was designed (see the following paragraph), and made available from April 30^th^ to June 8^th^, 2020. Both children/adolescents and parents were asked to complete the questionnaire, after expressing an informed consent. They were informed that the participation at the study was confidential. Data were collected anonymously (no name, nor contact information were allowed), as per Google’ privacy policy and treated for research purposes only. The voluntarily participation at the study was acknowledged with both a positive answer to a direct question and the submission of the questionnaire, that was not recorded if the participant did not complete it fully. The study was approved by the Ethics Committee of ASST dei Sette Laghi di Varese (n.82 of 2020), in compliance with the Declaration of Helsinki.

### The online questionnaire

The electronic survey was designed by a steering group of multidisciplinary scientists and academics (i.e. child neuropsychiatrists, psychologists and bioengineers) at the University of Insubria (principal investigator), following a structured review of the literature. The questionnaire included 38 single and multiple-choice questions. It started with a characterization section, that was addressed to parents only and investigated demographical and environmental factors. Besides the characterization of study participants, this section is useful to segment the population in different categories, that could have been impacted differently in different areas of difficulty. First, we hypothesize that the approach to the different aspects of life described by the endpoints could greatly vary according to age. Moreover, socioeconomic status could influence the availability of different instruments for virtual contacts or support and it is considered a crucial factor for development of cognitive functions, like attention, language and learning abilities (Schibli & D'Angiulli, 2013). Socioeconomic disadvantaged situations could also result in impossibility to attend remote classes (Agnelli Foundation, 2020), increased anxiety (Vazirani & Bhattacharjee, 2020) and violence against children (Cluver et al., 2020). The presence of siblings, instead, can affect the availability of resources and spaces for remote learning, but also have an impact on socialization (Szymańska, 2020). Family status (i.e. parents living together versus separate or single-parent families) can affect different psychological aspects and it is known that children of divorced parents may have social problems (Liu et al., 2000). The shift to homeworking was a never-experienced situation for many families and could impact life habits, but it could also be a chance for young children to be helped during remote classes. On the counterpart, having parents working in the healthcare field – that was the most exposed to the virus – could have increased children’s anxiety (Skokauskas et al., 2020). The latter could have been caused also by having contacts with people positive to COVID-19. Finally, it was possible to conjecture that the availability of a private outdoor space, such as a terrace or a garden, might have been helpful in getting some relief from the isolation (Jackson et al., 2021). A summary of these questions and available answers can be found in Table 1.

**Table 1:** Answers frequency divided by Controls and NDDs – stratifying variables

INDEPENDENT VARIABLES	CONTROLS	NDDs
Gender		
*Male*	3280	861
*Female*	3663	501
H-SES		
*1*	621	196
*2*	1442	329
*3*	1798	347
*4*	2324	372
*5*	758	118
Family Status		
*Separate/single-parent*	859	233
*Parents living together*	6084	1129
Siblings		
* 0*	1675	309
* 1*	4058	761
> *1*	1210	292
Outdoor		
*No*	701	160
*Yes*	6242	1202
Covid Family		
*No*	6648	1305
*Yes*	295	57
Covid Exposure		
*No*	6565	1268
*Yes*	378	94
School Grade		
*PS*	3318	455
*MS*	1790	442
*HS*	1835	465
School Class		
*PS-grade 1*	672	40
*PS-grade 2*	696	54
*PS-grade 3*	701	103
*PS-grade 4*	668	111
*PS-grade 5*	581	147
*MS-grade 1*	656	174
*MS-grade 2*	603	140
*MS-grade 3*	531	128
*HS-grade 1*	506	146
*HS-grade 2*	432	112
*HS-grade 3*	390	98
*HS-grade 4*	299	61
*HS-grade 5*	208	48
Remote Work		
*None*	3795	821
*One*	2328	423
*Both*	820	118
Healthcare Professional		
*None*	6157	1167
*One*	654	160
*Both*	132	35

Then, two corresponding set of questions were repeated, once addressed to parents and once addressed to children. These questions were selected to render the five areas of difficulties into the corresponding endpoints: *Remote Learning* (i.e. difficulties in attending online lessons and doing homework), *Lifestyle* (i.e. changing in lifestyle habits concerning sleep, diet and use of electronic devices), *Anxiety* (i.e. COVID-like symptoms, asking for information and reassurances about virus), *Sociality* (i.e. feeling of missing peers and school) and *Scolding* (i.e. possible increase in domestic disputes). The summary of these questions and available answers can be found in Table 2.

**Table 2:** Answers frequency divided by Controls and NDDs – endpoint question

		PARENTS		CHILDREN	
		Controls	NDDs	Controls	NDDs
REMOTE LEARNING	HOMEWORK				
	Continues doing homeworks	5221	669	4947	670
	Stopped/has difficulties	1722	693	1996	692
	REMOTE CLASS				
	Attending regularly	5540	809	5275	788
	Not attending/having difficulties	1403	553	1668	574
LIFESTYLE	DIETARY STYLE				
	More vegetables/ unchanged	4067	741	3878	701
	Changed otherwise	2876	621	3065	661
	SLEEP HABITS				
	Unchanged	883	186	785	172
	Changed	6060	1176	6158	1190
	NIGHT WAKING				
	Absent/decreased	5896	1130	5852	1067
	Increased	1047	232	1091	212
	ELECTRONIC DEVICES				
	Unchanged/slightly changed	6263	1204	6292	1204
	Changed	680	158	651	158
ANXIETY	INFO COVID-19				
	Never/seldom	5833	1165	5913	1173
	Often/everyday	1110	197	1030	189
	SYMPTOMS				
	Never/seldom	6887	1343	6850	1334
	Often/every day	56	19	93	28
	REASSURANCES				
	Never/seldom	6700	1299	6773	1316
	Often/every day	243	63	170	46
	NEW PAIN				
	No	6455	1212		
	Yes	488	150		
SOCIALITY	ASKS FOR SCHOOL				
	Sometimes/often			5125	861
	Never			1818	501
	MISSES MATES				
	A bit/a lot			6748	1297
	No			195	65
SCOLDING	SCOLDING				
	As usual/less often	4667	923	4721	905
	More often	2276	437	2222	454

In total, 26 questions were directed to parents and 12 to children. When children were less then twelve years old, parents were suggested to help them in questionnaire administration; parental support for children under the age of 12 is also useful for children whose diagnosis could influence reading comprehension (e.g. learning or language disorders).

### Data analysis

Statistical analysis was conducted using R (version 4.0.1). For all tests, significance was set at 5%. The answers of the characterization section (first part), answered by parents only, were used as independent variables. The answers were both ordinal and categorical. The latter were always 2-levels answers, therefore could be treated as binary variables.

Specifically:

- NDD: certified NDDs [2-levels: control (0); NDD (1)];
- Gender: [2-levels: male (0); female (1)];
- H-SES: socioeconomic status through Hollingshead index (Hollingshead, 1975), [5-levels: from very low to very high];
- Family status: [2-levels: parents living separated or single-parent family (0); parents living together (1)];
- Presence of siblings: [3-levels: none, one, more than one];
- Attended school: [3-levels: PS, MS, HS];
- Attended school class: [13-levels: from 1^st^grade PS to 5^th^grade HS];
- Presence of COVID-19 cases in the family: [2-levels:no (0), yes (1)];
- Exposure to COVID-19 cases outside the family: [2-levels:no (0), yes (1)];
- Presence of outdoor spaces at home: [2-levels:no (0), yes (1)];
- Parents working as healthcare professionals: [3-levels: none, one, both];
- Parents working from home: [3-levels: none, one, both].

The answers of the endpoints’ sections were dichotomized to give them a positive (1) or negative (0) meaning, according to the effect we hypothesized on the endpoints itself. Then, for the endpoints formed by more than one question (*Remote Learning, Lifestyle, Anxiety, and Social*), the answers were transformed using Item Response Theory (IRT). Such transformation allows to represent a set of items as a unique latent trait, which measures a subject’s attitude on a specific topic (the endpoint). Given the consistent attribution of the value 1 to positive answers, the IRT transformation produced an ordinal variable with high values corresponding to positive attitude on the endpoint. After IRT, if the granularity of the latent trait was above 5, the levels of the latent trait were additionally reduced to obtain a 3-point scale (low, medium, and high values). The 33^rd^ and the 66^th^ percentiles were chosen to distinguish between levels. The obtained endpoints are reported in Table 3. As for the primary objective, to assess the effect of different factors on the endpoints, logistic regression was adopted. For ordinal endpoints (*Remote Learning*; *Lifestyle*; *Anxiety*; *Sociality*), we applied an ordinal logistic regression, while we opted for a binary logistic regression for binary endpoints (*Scolding*). For both ordinal and logistic regressions, we first checked the assumptions of absence of multicollinearity between independent variables through the variance inflation factor (VIF), verifying that there was not multicollinearity in the dataset (VIF below 10) (Hair et al., 2010). For the ordinal logistic regression, we also verified the respect of proportional odds using the Brant Test. When a variable violated the assumption of proportional odds, it was removed from the set of independent variables for the ordinal logistic regression models. After that, ordinal logistic regression and binary logistic regression models have been fitted, considering ordinal and binary endpoints, respectively, as dependent variables. In a second step, we further selected the best subset of covariates through the Akaike Information Criterion AIC-based step forward and backward procedure. To conclude, all the non-significant variables were excluded and only the remaining ones were used to build the final model, and finally assess the influence of each independent variable on the endpoint. However, the NDD group was composed by different sub-types, that might differently affect the endpoint. With the aim of assessing the effect of specific NDD type on the endpoints, we considered only subjects who did not present a comorbidity with another NDD type, and performed a Kruskal–Wallis test, considering the ordinal endpoint as dependent variable, and the NDD type or control condition as independent variable. When significance was reached, Mann–Whitney U test with Bonferroni correction was leveraged as post hoc. Given the low sample size of the single NDD groups in respect to the controls, we computed the effect size with Cohen’s d. Concerning the secondary objective, to test the agreement between parents and students, we computed the Fleiss Kappa on the binarized questions which composed the endpoints. Fleiss Kappa is an index of agreement adaptable in case of multiple raters. The agreement was computed for all subjects and separately for NDDs and Controls. To investigate between-group differences in agreement homogeneity (NDDs and Control) we also ran a homogeneity score test (Honda & Ohyama, 2020). To assess the potential influence of parents’ presence during questionnaire completion for children younger than 12 years old, we applied the same homogeneity test between children and adolescents groups, using the second year of middle school as a cutoff.

**Table 3:** Questions considered to build the endpoint and meaning of the endpoint itself

ENDPOINT	ANSWERED BY	TYPE/GRANULARITY	ITEMS TO MESURE THE ENDPOINT	MEANING/DIRECTION
*Remote Learning*	Children	4-level ordinal	-Difficulties with remote classes -Difficulties with homework	-Was it difficult to follow remote school classes? -High values: low difficulties
	Parents			
*Lifestyle*	Children	3-level ordinal	-Dietary style -Sleep habits -Night waking -Exposure to electronic devices	-Did daily lifestyle change with lockdown? If so, to which extent and how? -High values: reduced change
	Parents			
*Anxiety*	Children	3-level ordinal	-Info about COVID-19 -COVID-19-like symptoms -Reassurances seeking about one's own health	-Did anxiety signs appeared or changed with COVID-19 pandemic? -High values: low anxiety
	Parents			
*Sociality*	Children	4-level ordinal	-Missing peers	-Has the lack of social relationships been perceived as negative? -High values: high feeling of missing peers
*Scolding*	Children	Binary	-Scolding increased	-Did children show difficulties in coping with COVID-19 lockdown, resulting in frequent scolding? -High values: rarely scolded
	Parents			

## Results

**Fig. 1:**
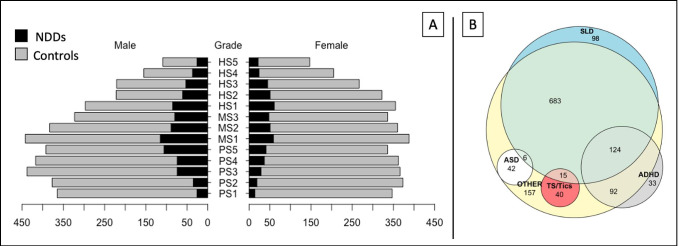
Population description. A: pyramid plot of subjects’ distribution among gender, school grade and NDDs/Control condition. B: Euler-Venn diagram of NDDs distribution. The overlapping parts represent comorbidities

8305 questionnaires were analyzed. Subjects’ details are reported in Table 1 and in Fig. 1A, where students are arranged by gender, school grade and NDDs category or control group. Specifically, the sample comprised 4141 males and 4164 females. 45.4% of the respondents attended primary school (median age (inter quartile range): 9(2) years old), 26.9% attended middle school (13(2) years old), 27.7% (17(3) years old). The median socioeconomic status, in a range 1–5 (Hollingshead, 1975), was 3, with a 2-points symmetric inter quartile range. Students affected by NDDs were 1362 (861 males, 501 females); their distribution in NDDs type is reported in Fig. 1B. Specifically, 983 were affected by SLD, 313 by ADHD, 72 by TS/Tics, 48 by ASD, 1159 by other NDDs. The overlaps between areas represent the presence of a comorbidity between different NDD.

### Answer distribution

Answer frequency is reported in Table 1 (A for characterization, and B for endpoints), divided by children and parents’ questions, and stratified by NDDs or Controls condition. Thanks to the parents’ help, we did not have missed answers in the children sample, even for children with NDD.

### Logistic regression and post hoc

Results of the logistic regression are summarized in Table 4, whilst the post hoc tests are reported in Online Resource 1. In the post hoc, given the high degree of comorbidities between the OTHER condition and the remaining NDD types, to assess the effect of the single NDD on the endpoint, it was decided to disregard the comorbidity with the OTHER, and to delete only children who had comorbidities between the remaining NDD types. Therefore, the number of children in each NDD type was: 781 in SLD, 125 in ADHD, 46 in TS/Tics, 42 in ASD. In Table 4, for each model, the significant predictors are presented in order of t-value. The algebraic sign of the value represents the direction of the effect over the endpoints, given the coding of the independent variables previously described. The comment below each numerical result is intended to help interpretation. As shown, the presence of NDDs has a strong negative effect on the *Remote Learning* endpoint, for both students and parents, suggesting that the presence of NDDs made it more difficult for students to attend school remotely, compared to the control group. The post hoc analysis confirmed the result, as most of the comparisons between the control group and the specific NDDs groups showed a medium to large effect. Between-NDDs comparisons were never significant. The analysis reports that remote learning difficulties were starker, especially for children. Family status impacted this endpoint in several ways: problems arose in families with lower socio-economic background; in those where only one parent was present; in those where parents worked in the healthcare field; in those where there were many siblings. On the contrary, the presence of an outdoor space was a facilitator, together with not having any contact with people with COVID-19. A significant (negative) effect of NDDs was also reported for the *Sociality* endpoint, meaning that subjects with NDDs tended to be missing their peers less. On the contrary, children without siblings miss more their friends. As for the *Scolding* endpoint, children/adolescents with NDDs felt like they were scolded more often by their parents during the COVID-19 lockdown. This endpoint was affected from the attended school, as younger children were scolded more; from the family composition, as children with separated parents were scolded more; from the presence of an outdoor space, as those constrained at home were scolded more. Concerning the *Anxiety* endpoint, the analysis suggested that parents of NDDs children perceived their kids slightly more stressed and anxious compared to parents of controls. This was mainly due to the ASD group, which differed from the others with medium effect size (additional data are given in Online Resource 1). Children who had contacts with people positive to COVID-19 suffered more from anxiety, whilst an outdoor space had a beneficial effect. Regarding the *Lifestyle* endpoint, we did not find any difference between NDDs and controls, but we observed a higher change in lifestyle of adolescents; in those who came from a family with low socioeconomic status; to those where parents started working from home; and where an outdoor space was not available.

**Table 4:** Logistic regression results. For each model, the significant predictors are presented in order of t-value. The algebraic sign of the value represents the direction of the effect over the endpoint

	CHILDREN					PARENTS				
	Ordered Significant Covariates	value ± standard error	t-value	OR	p-value	Ordered Significant Covariates	value ± standard error	t-value	OR	p-value
REMOTE LEARNING	NDD	-0.967±0.058	-16.721	0.381	<0.0001	NDD	-1.191±0.059	-20.171	0.304	<0.0001
		*The NDD condition negatively impacted Remote Learning*					*The NDD condition negatively impacted Remote Learning*			
	School class	0.084±0.007	12.316	1.087	<0.0001	H-SES	0.161±0.020	7.920	1.175	<0.0001
		*Older children had less difficulties in Remote Learning*					*Children with high socio-economic status had less difficulties in Remote Learning*			
	H-SES	0.166±0.020	8.502	1.181	<0.0001	Siblings	-0.538±0.072	-7.431	0.584	<0.0001
		*Children with high socio-economic status had less difficulties in Remote Learning*					*Having more siblings negatively impacted Remote Learning*			
	Siblings	-0.495±0.069	-7.152	0.609	<0.0001	Family status	0.374±0.067	5.575	1.454	<0.0001
		Having more siblings negatively impacted Remote Learning					Having both parents was beneficial for Remote Learning			
	Outdoor	0.402±0.070	5.729	1.495	<0.0001	Outdoor	0.321±0.073	4.425	1.378	<0.0001
		*Outdoor space availability was beneficial for Remote Learning*					*Outdoor space availability was beneficial for Remote Learning*			
	Family status	0.284±0.065	4.365	1.329	<0.0001	School class	0.069±0.019	3.680	1.071	<0.001
		*Having both parents was beneficial for Remote Learning*					*Older children had less difficulties in Remote Learning*			
	Healthcare professionals	-0.290±0.116	-2.504	0.748	0.013	Healthcare professionals	-0.377±0.120	-3.153	0.686	0.002
		*Having parents working in the healthcare negatively impacted Remote Learning*					*Having parents working in the healthcare negatively impacted Remote Learning*			
	COVID-19 exposure	-0.233±0.099	-2.359	0.792	0.018	School	0.173±0.076	2.275	1.189	0.023
							Older students had less difficulties in Remote Learning			
		*Having contacts with people affected by COVID-19 negatively impacted Remote Learning*				COVID-19 exposure	-0.200±0.102	-1.971	0.818	0.049
							*Having contacts with people positive to COVID-19 negatively impacted Remote Learning*			
LIFESTYLE	School class	-0.050±0.006	-7.974	0.951	<0.0001	School class	-0.057±0.006	-9.237	0.944	<0.0001
		*Older children changed their Lifestyle more*					Older children changed their *Lifestyle* more			
	Outdoor	0.378±0.072	5.271	1.459	<0.0001	Outdoor	0.454±0.070	6.462	1.575	<0.0001
		Outdoor space availability was associated with less changes in *Lifestyle*					*Outdoor space availability was associated with less changes in Lifestyle*			
	H-SES	0.079±0.022	3.680	1.082	<0.001	H-SES	0.111±0.018	6.010	1.117	<0.0001
		Children with high socio-economic status changed their *Lifestyle* less								
	Remote work	-0.169±0.071	-2.367	0.844	0.018		*Children with high socio-economic status changed their Lifestyle less*			
		*Children change their Lifestyle more if parents performed remote working*								
ANXIETY	COVID-19 family	-0.457±0.130	-3.527	0.633	<0.001	COVID-19 exposure	-0.776±0.186	-4.168	0.460	<0.0001
		*Having cases of COVID-19 among family members was associated with more Anxiety*					*Knowing people affected by COVID-19 was associated with more Anxiety*			
	Outdoor	0.253±0.091	2.774	1.288	0.006	Outdoor	0.439±0.156	2.811	1.551	0.005
		*Outdoor space availability was associated with less Anxiety*					*Outdoor space availability was associated with less Anxiety*			
	School	-0.214±0.097	-2.206	0.807	0.027	COVID-19 family	-0.577±0.217	-2.655	0.561	0.008
		*Children attending higher school orders had more Anxiety*					*Having cases of COVID-19 among family members was associated with more Anxiety*			
	School class	0.052±0.024	2.180	1.053	0.029	School class	0.039±0.017	2.346	1.040	0.019
							*Older children had less Anxiety*			
		*Older children had less Anxiety*				NDD	-0.306±0.140	-2.184	0.737	0.029
							*Having a NDD was associated with more Anxiety*			
SOCIALITY	School class	-0.175±0.020	-8.772	0.839	<0.0001					
		*Younger children missed more their peers*								
	NDD	-0.393±0.063	-6.258	0.675	<0.0001					
		*Children with NDD missed less their peers*								
	Siblings	-0.267±0.076	-3.512	0.765	<0.001					
		*Children who have siblings missed less their peers*								
	Family status	0.222±0.071	3.154	1.249	0.002					
		*Students who do not have both parents at home missed less their peers*								
	School	0.229±0.078	2.870	1.257	0.004					
		*Children attending higher school orders missed less their peers*								
SCOLDING	School class	0.143±0.008	18.856	1.153	<0.0001	School class	0.195±0.008	24.537	1.215	<0.0001
		*Older children were associated with less Scolding*					*Older children were associated with less Scolding*			
	Sex	0.207±0.049	4.251	1.230	<0.0001	Sex	0.324±0.050	6.534	1.382	<0.0001
		*Female were associated with less Scolding*					*Female were associated with less Scolding*			
	COVID-19 exposure	-0.302±0.102	-2.979	0.739	0.003	Outdoor	0.226±0.078	2.886	1.254	0.004
		*Children who had contacts with people affected by COVID-19 were associated with more Scolding*					*Outdoor availability was associated with less Scolding*			
	NDD	-0.184±0.066	-2.796	0.832	0.005	Family status	0.16 ±0.073	2.245	1.178	0.025
		*Children affected by NDDs were associated with more Scolding*								
	Outdoor	0.174±0.078	2.243	1.190	0.025		*Children with both parents at home were associated with less Scolding*			
		*Outdoor availability was associated with less Scolding*								

### Child-parent agreement

**Fig. 2:**
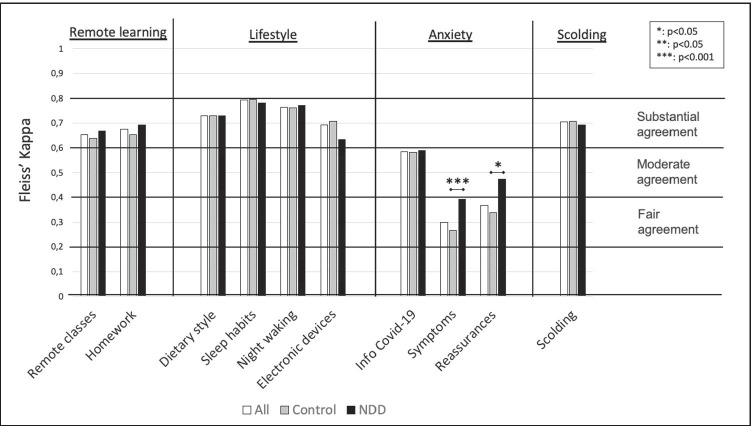
Fleiss’ Kappa for the agreement. Each bar represents the agreement on an item, considering all respondents together (white bars), Controls only (grey bars), or NDDs only (black bars), grouped by endpoint. Agreement thresholds are reported on the right. Asterisks indicate a significant group effect in agreement homogeneity

Figure 2 reports the results for the agreement on each item, grouped by endpoint. Agreement thresholds are reported on the right. Substantial agreement (0.6 < *K* < 0.8, *p* < 0.001) arose from questions concerning *Remote Learning*, *Lifestyle*, and *Scolding*. In the *Anxiety* endpoint, a moderate agreement (0.58) was reached concerning the request of information about the virus. The feeling of COVID-like symptoms and the request for reassurances reached a fair level of agreement (0.30 and 0.37, respectively). The *Sociality* endpoint included questions directed to children only, therefore agreement could not be computed. In regard to homogeneity in agreement between NDDs and Controls, a significant group effect was found in questions related to the *Anxiety*, i.e. Symptoms (*p* < 0.001) as the NDD group obtained a greater value than controls (*p* < 0.001, *K* = 0.393, *K* = 0.298, respectively); in Reassurances (*p* = 0.017), a fair agreement was found in Controls (*K* = 0.339), whilst NDDs showed a moderate agreement (*K* = 0.474). As for the potential influence of parents’ during questionnaire completion for children younger than 12 years old, the homogeneity test did not produce a significant effect in seven out of ten questions. Significance was reached in two items of the *Lifestyle* endpoint, i.e. *Dietary style* (*p* = 0.013, children’s *K* = 0.71, adolescents’ *K* = 0.75), *Night awakening* (*p* = 0.044, children’s *K* = 0.74, adolescents’ *K* = 0.78); and one item of the *Anxiety* endpoint, i.e. *Info Covid-19* (*p* = 0.043, children’s *K* = 0.65, adolescents’ *K* = 0.62). In these three questions, the agreement was always slightly higher between parents and adolescents, rather than between parents and children.

## Discussion

In this study, the psycho-social impact of COVID-19 on children and adolescents with and without NDDs was investigated through an online questionnaire; the answers given by parents and children/adolescents were paired up—and then compared—in order to ensure reliable agreement. The aim of the study was to understand the effect of NDD condition, socio-demographic status, familiar and home environment and COVID-19 exposure on children/adolescent everyday lives. Such impact was evaluated considering five endpoints: *Remote Learning* difficulties, *Lifestyle* modifications, level of virus-related *Anxiety*, *Social* relationships and trend of familiar disputes—operationalized as *Scolding*. In what follows, we discuss the effect of independent variables on each dependent variable (AKA endpoint). We paid special attention on NDD effect, hypothesizing that COVID-19 lockdown should be even more complicated for these children; besides, we have discussed the other environmental variables with respect to the difficulties encountered by all children.

### Remote learning

The COVID-19 pandemic has produced one of the largest breakdowns of education system in contemporary age. The United Nation has claimed that it has been a global crisis of teaching and learning. Closures of schools and educational structures have impacted 94% of the world’s student population; the emergency has exacerbated preexisting disparities by reducing the learning opportunities for many vulnerable children (United Nations, 2020). Indeed, our analysis reveals a disadvantage in remote learning for children belonging to families with low socio-economic status. This finding is supported by a previous Italian study (Agnelli Foundation, 2020), which shows that even proper methods and technologies would make little sense in socio-culturally disadvantaged contexts. Moreover, we found that the presence of siblings had a negative effect on lessons and homework; it may be related to the fact that the only children usually receive larger amounts of feedbacks and attention from their parents and this is assumed to strengthen their self-confidence (Cao et al., 2021), improving their school performance as a result. Furthermore, availability of technology devices and quiet environments are supposed to be more difficult to find in large families and/or little homes, making attention harder to keep. In addition, children at their first years of school struggled more with remote learning than their older colleagues, for at least three reasons: (i) they probably are less confident with technology; (ii) there is an intrinsic difficulty in translating PS lessons into online sessions; (iii) most children could not pay attention as long as older students did. The psychological effect of COVID-19 is clear-cut: stress responses are more frequent in children of healthcare workers, because they probably realize their parents are putting themselves at risk to be infected by or to die of COVID-19 (Skokauskas et al., 2020). Regarding NDDs, our analysis suggests that the presence of such pathological condition had a strongly negative effect on the *Remote learning* endpoint, meaning that children with NDDs suited distance learning worse than controls. Our finding is supported by international studies, that show difficulties in attending online lessons by children with special needs; they actually have to face barriers such as the absence of necessary equipment and internet access, but most of all they had no appropriate assistance to follow the online programs (United Nations, 2020). In point of fact, children with NDDs require individualized education plans and remote learning could not always be afforded to them. With the abrupt shift of caretaking responsibilities exclusively on the family unit, and without the support that schools typically provide, the burden was overwhelming for many parents (Tajè et al., 2020). In accordance with our results, another Italian study shows that, despite the efforts of the schools, during remote learning at least 1 out of every 4 disabled students got left behind (Agnelli Foundation, 2020). School typically offers to children some sense of freedom, through the opportunity of interaction with peers and teacher, besides providing learning contents; furthermore, it plays an edifying role in promoting physical activity, healthy and social habits (Ghosh et al., 2020). According to that, difficulties in attending remote learning, especially for children with NDD, causes much more than loss of learning opportunities.

### Lifestyle

We found no significant difference in lifestyle changes during the lockdown in NDDs and control group; nevertheless, we found drastic changes in the lifestyles of both groups, especially in the adolescents, who were probably freer to change their daily routine than the younger. We investigated diets, sleeping habits and usage of electronic devices and detected changes of usual habits in all these areas. Concerning diets, our findings are supported by previous researches: Italians’ food habits changed and the weight increased in 48.6% of the population (Di Renzo et al., 2020). We also observed changes in sleep routine: children/adolescents started going to sleep later and waking up later. The drawbacks of this time shift are the reduced exposure to sunlight and the increased time spent working in bed, resulting in potential troubles in falling asleep (Becker & Gregory, 2020). Noticeably, families where parents could work from home had a greater modification in habits, and it could be due to a generalized change in time organization. Moreover, we observed an augmented usage of electronic devices, for both entertainment and educational purposes. The use of internet has increased dramatically during the COVID-19 pandemic. For young people, internet is appealing and quick to access and may be an easy way to relieve stress, escaping from reality; on the other hand, excessive users will be more focused on Internet and less interested in real life. The WHO “COVID-19:24/7 parenting” guide recognizes that web usage is essential to adolescent’s is sociability, but suggests that the content should be carefully monitored by parents (WHO et al., 2020).

### Sociality

As social distancing seems to be effective in reducing the transmission of COVID-19, the *Sociality* endpoint was made up to understand how children reacted to the deprivation of their mates and school life. We found that older children missed their peers less and this could probably be due to the diffused use of media amongst adolescents during the last decade, regardless of COVID-19 lockdown. In addition, family environment appeared as fundamental for sociality; we found indeed that the presence of siblings reduced the lack of friends, confirming that siblings play a major role in human relationships and that children often perceive them as one of the most important people in life (Szymańska, 2020). Children of families with divorced parents missed their friends less, too; however, this could be discussed according to socialization problems and high restraint that may occur in such children (Liu et al., 2000), sometimes making them entangled in closer relationships with the single parent and/or developing internalizing attitudes (Cohen & Weitzman, 2016). Results eventually show a significant difference between NDDs and controls: children with NDDs stated they miss their peers a little. We could consider different features of NDDs to explain this finding. For example, children with ASD have, by definition, persistent deficits in social communication and social interaction across multiple contexts (American Psychiatric Association, 2013); this could explain their reduced interest in keeping relationships during the lockdown period. Moreover, it is known that patients with TS/TICS and ADHD frequently experience feelings of loneliness and peer rejection (Cox et al., 2019), being therefore accustomed to having less contact with peers regardless of the pandemic. On this topic, the American Psychological Association published practical advices for caregivers of children with disabilities during the time of COVID-19, suggesting to contact others virtually in order to cope with self-isolation (American Psychological Association, 2020).

### Anxiety

The spread of the COVID-19 pandemic has led countries to adopt restrictions in order to safeguard the health of citizens; such restrictions may have negative psychological consequences as people are limited in their social and leisure activities, thus daily life stressors could be harder to bear with (Niziurski & Schaper, 2021). Therefore, understanding children’s reactions and emotions is essential to properly address their needs. In our study, we found that the most important predictor of anxiety was having a direct contact with people affected by COVID-19. This group of children requested more information about virus and needed more reassurance about their health. In fact, it is predictable that, during a global pandemic, children may develop feelings of sadness, anxiety, fear of death and of their parents’ death, with a detrimental effect on their psychological well-being (Singh et al., 2020). Conversely, the availability of an outdoor space is a factor that can mitigate fear: the availability of outdoor spaces has been proven to have positive effects on stressors, improving physical well-being and quality of life, especially in cases of forced home confinement (Jackson et al., 2021). A significant difference between NDDs and control group was found for *Anxiety* endpoint as well. Parents of children with NDDs perceived their kids slightly more stressed and anxious compared to controls; children with NDDs did the same, reporting a high self-perception of stress and anxiety. The post hoc analysis showed that all NDDs but ASD are more anxious than controls. These results could be explained by recurrent comorbidities between most NDDs and anxiety disorders (D’Agati et al., 2019; Haft et al., 2019). It is clear that understanding the response to anxiety is a crucial step also to optimize policy implementation in a crisis; in a previous Indian study (Vazirani & Bhattacharjee, 2020) investigating the correlation between anxiety, health, economic conditions and lack of food during lock-down from COVID-19, it was found that understanding the response to anxiety is fundamental when pandemics create limitation in the individual’s ability to mitigate the problem. Therefore, in order to prevent and face anxiety and fear in children, healthcare professionals spoke to them about COVID-19 and encouraged them to talk about their feelings (American Psychiatric Association, 2020), according to operational indications about clinical-assistance activities, drafted by the Italian Society of Infancy and Adolescence Neuropsychiatry (SINPIA, 2020).

### Scolding

We asked parents and children about their perception of family dynamics during lockdown, to explore comprehensive distress level. In particular, we asked parents and kids how often they scolded or were scolded, respectively and we found a general increase of about 30% in family disputes. In fact, there is compelling evidence in literature on domestic violence increase in families during lockdown caused by health emergencies (Pereda & Dìaz-Faes, 2020). The diffuse economic crisis was an additional risk factor for domestic abuse and violence against children (Cluver et al., 2020). During the first Italian lockdown, that lasted about 2 months, families experienced high levels of distress and fear—emphasized by medias—that led to a challenge about their tolerance level and empathy. Similar to findings concerning *Anxiety*, we found that the presence of an outdoor space gives relief to familial tension, in accordance with the common belief that nature has positive impacts on children's health, including physical, mental and social dimensions (Tillmann et al., 2018). Our results show a significant difference between NDDs and control groups also in *Scolding* endpoint: children with NDDs stated they were scolded more often during lockdown. This could be explained with the increase of behavioral problems throughout home-confinement; the unexpected lifestyle changes during the lockdown—together with augmented parental stress and NDD’s condition per se—could in fact break up the emotional and behavioral balance in children.

### Child-parent agreement

In our study, we considered: (a) agreement between parents and children and (b) the effect of NDD condition on the agreement. We found substantial agreement between all parents and children (a) for the endpoints other than *Anxiety*, in which K index was slightly lower. It may be due to the fact that anxiety is internalizing by nature, so it could be difficult to investigate it objectively. ASD and TS groups mainly accounted for the lower agreement, confirming that, in ASD subjects, anxiety is a dominant presenting problem, even though some autistic dimensions—most of all the communication deficit—prevent children to overtly talk about their feelings (American Psychiatric Association, 2013). TS subjects are indeed known to have poor internalizing symptoms and predominant externalizing ones (Ghanizadeh & Mosallaei, 2009); their parents could not properly identify their real worries, their attention being focused on other behaviors, such as movements and vocalizations. A similar trend of agreement about anxiety was noted in previous studies on TS, too (Cavanna et al., 2013). The higher agreement detected in the NDD group with respect to controls (b) could be discussed referring to the larger amount of time spent together by children with special needs and their parents. Caregivers are thus emotionally closer to their kids, given the fact that they offer them constant support for everyday activities, probably becoming more empathic and familiar with their feeling (Hohlfeld et al., 2018). Additionally, we found that parents did not influence children (< 12 years old), when helping them in questionnaire completion. In fact, when an age effect emerged, agreement between adolescents and their parents was stronger than agreement between children and their parents. This possibly suggests a difficult self-perception in children or even a high parental apprehension, but not an acquiescence bias (Cabitza et al., 2019).

## Limitations

It must be considered that children with NDDs usually cope harder with learning, anxiety, routine and sociality even before pandemic. Although questions were purposed to compare the pre-pandemic situation to the current one, it was not possible to depict a baseline situation (i.e. before COVID-19). In addition, even if the agreement homogeneity analysis supports that parents’ influence on children fulfilment was minimal, some kind of influence may have occurred. Some students younger than 12 years old could have completed the questionnaires alone; on the other hand, those with reading problems, language or intellectual disabilities could have received help also at older ages. This complex situation makes it hard to properly assess the presence of the acquiescence bias in answers. Finally, we selected a set of independent variables driven by literature and experience, but it is possible that other factors have latently influenced the endpoints. As an example, other similar studies investigate if families live in an urban or rural context, but the substantial territorial homogeneity of the Varese province makes it less meaningful than having a garden, in combination with the socioeconomic status. Moreover, adding more independent variables would have reduced the power of the analysis.

## Conclusions

The first Italian COVID-19 lockdown had a great impact on families. In this study, children and adolescents with NDDs showed a more significant impact in remote learning, behavioral and emotional aspects (i.e. they were scolded more and were anxious, they missed their peers less), compared to controls. Our results could provide useful information for local policy makers, school authorities, health services and families in order to implement the best support strategies for children and adolescents with NDDs in the event of a re-occurrence of a pandemic or a situation of environmental contamination and general restrictions. Regarding remote learning, it is essential to provide personalized solutions adapting to the needs and abilities of children and they should be specifically tailored to NDDs. In particular, a constant collaboration between families and teachers should be guaranteed eventually involving a 1:1 remote support from special aid teachers. Specific training for teachers could be promoted, aiming at a better didactical and psychological support. Mental health services for children and adolescents should ensure continuity of care using telemedicine tools to ensure the necessary support and avoid the risk of contagion from COVID-19. The ESSENCE European project (ESSENCE, 2020) addresses most of these needs, offering a telemonitoring and teleassistance platform to support children, both with and without specific difficulties. It acts as a facilitator for children and families to keep in touch with peers, teachers and, eventually, clinical services. Furthermore, the importance of encouraging the preservation of social and familiar relationships is clear, to prevent their worsening in relational impairment. Finally, special permission and safety measures should be accorded to let children play outdoor, with significant benefits on both their physical and mental health.

## Supplementary Information

Below is the link to the electronic supplementary material.

Supplementary file1 (DOCX 72 KB)

## Data Availability

the datasets generated during and/or analyzed during the current study are available from the corresponding author on reasonable request.
