# Supplementary material for: Investigating the effects of COVID-19 lockdown on Italian children and adolescents with and without neurodevelopmental disorders: a cross-sectional study
Source: Curr Psychol. 2021 Oct 25;42(10):8615–31. doi: 10.1007/s12144-021-02321-2 (PMC8542499; doi:10.1007/s12144-021-02321-2)
Supplement: Supplementary file 1 — Supplementary file1 (DOCX 72 KB) [file 12144_2021_2321_MOESM1_ESM.docx]

| **ONLINE RESOURCE 1** | | | | | | | | | | | | | | |
| --- | --- | --- | --- | --- | --- | --- | --- | --- | --- | --- | --- | --- | --- | --- |
| *Post Hoc analysis for between-groups differnces in the Endpoints* | | | | | | | | | | | | | |  |
|  |  |  | **CHILDREN** |  |  |  | |  |  |  | **PARENTS** |  |  |  |
| ***Remote learning*** | | | | | | ***Remote learning*** | | | | | | | | |
|  | **CONTROL** | **ASD** | **ADHD** | **SLD** | **TS/TICS** |  |  | | **CONTROL** | **ASD** | **ADHD** | **SLD** | **TS/TICS** |  |
| **median (IQR)** | 0.308 (0.866) | -0.434 (1.405) | -0.558 (1.405) | -0.434 (1.405) | -0.558 (1.405) |  |  | | 0.233 (0.785) | -0.645 (1.401) | -0.645 (1.401) | -0.552 (1.401) | -0.645 (1.401) |  |
| **CONTROL** |  | p=0.014, d=0.526 (medium) | p<0.001, d=0.671 (medium) | p<0.001, d=0.419 (small) | p=0.012, d=0.510 (medium) |  | **CONTROL** | |  | p<0.001, d=0.901 (large) | p<0.001, d=0.761 (medium) | p<0.001, d=0.521 (medium) | p<0.001, d=0.835 (large) |  |
| **ASD** |  |  |  |  |  |  | **ASD** | |  |  |  |  |  |  |
| **ADHD** |  |  |  |  |  |  | **ADHD** | |  |  |  |  |  |  |
| **SLD** |  |  |  |  |  |  | **SLD** | |  |  |  |  |  |  |
| **TIC/TS** |  |  |  |  |  |  | **TIC/TS** | |  |  |  |  |  |  |
| ***Lifestyle*** | | | | | | ***Lifestyle*** | | | | | | | | |
|  | **CONTROL** | **ASD** | **ADHD** | **SLD** | **TS/TICS** |  |  | | **CONTROL** | **ASD** | **ADHD** | **SLD** | **TS/TICS** |  |
|  | 0.143 (0.697) | 0.338 (0.697) | -0.265 (0.697) | -0.265 (0.697) | -0.265 (0.697) |  |  | | 0.193 (0.842) | 0.277 (0.842) | -0.137 (0.842) | 0.117 (0.842) | -0.137 (0.842) |  |
| **CONTROL** |  |  |  |  |  |  | **CONTROL** | |  |  |  |  |  |  |
| **ASD** |  |  |  |  |  |  | **ASD** | |  |  |  |  |  |  |
| **ADHD** |  |  |  |  |  |  | **ADHD** | |  |  |  |  |  |  |
| **SLD** |  |  |  |  |  |  | **SLD** | |  |  |  |  |  |  |
| **TIC/TS** |  |  |  |  |  |  | **TIC/TS** | |  |  |  |  |  |  |
| ***Anxiety*** | | | | | | ***Anxiety*** | | | | | | | | |
|  | **CONTROL** | **ASD** | **ADHD** | **SLD** | **TS/TICS** |  |  | | **CONTROL** | **ASD** | **ADHD** | **SLD** | **TS/TICS** |  |
|  | 0.115 (0) | 0.115 (1.128) | 0.115 (0) | 0.115 (0) | 0.115 (0) |  |  | | 0.158 | 0.158 (1.603) | 0.158 (0) | 0.158 (0) | 0.158 (0.681) |  |
| **CONTROL** |  | p=0.047, d=0.595 (medium) |  |  |  |  | **CONTROL** | |  | p=0.020, d=0.696 (medium) |  |  |  |  |
| **ASD** |  |  |  | p=0.020, d=-0.585 (medium) | p=0.023, d=-0.715 (medium) |  | **ASD** | |  |  |  | p=0.037, d=-0.663 (medium) |  |  |
| **ADHD** |  |  |  |  |  |  | **ADHD** | |  |  |  |  |  |  |
| **SLD** |  |  |  |  |  |  | **SLD** | |  |  |  |  |  |  |
| **TIC/TS** |  |  |  |  |  |  | **TIC/TS** | |  |  |  |  |  |  |
| ***Sociality*** | | | | | |  | | |  |  |  |  |  |  |
|  | **CONTROL** | **ASD** | **ADHD** | **SLD** | **TS/TICS** |  |  | |  |  |  |  |  |  |
|  | 0.157 (1.078) | 0.157 (1.078) | 0.157 (1.078) | 0.157 (1.078) | 0.157 (1.078) |  |  | |  |  |  |  |  |  |
| **CONTROL** |  |  |  | p<0.001, d=0.296 (small) |  |  |  | |  |  |  |  |  |  |
| **ASD** |  |  |  |  |  |  |  | |  |  |  |  |  |  |
| **ADHD** |  |  |  |  |  |  |  | |  |  |  |  |  |  |
| **SLD** |  |  |  |  |  |  |  | |  |  |  |  |  |  |
| **TIC/TS** |  |  |  |  |  |  |  | |  |  |  |  |  |  |
